# Influence of Deuterium-Depleted Water on the Isotope D/H Composition of Liver Tissue and Morphological Development of Rats at Different Periods of Ontogenesis

**DOI:** 10.29252/.23.2.129

**Published:** 2019-03

**Authors:** Alexandr Alexandrovich Basov, Аnna Anatolyevna Elkina, Andrei Alexandrovich Samkov, Nikita Nikolaevich Volchenko, Arcadii Victorovich Moiseev, Liliya Viacheslavovna Fedulova, Mikhail Gennadievich Baryshev, Stepan Sergeevich Dzhimak

**Affiliations:** 1 Kuban State Medical University, Krasnodar, Russian Federation, Russia;; 2 Kuban State University, Krasnodar, Russian Federation, Russia;; 3 Kuban State Agrarian University, Krasnodar, Russian Federation, Russia;; 4 The V.M. Gorbatov Federal Research Center for Food Systems of Russian Academy of Sciences, Moscow, Russian Federation, Russia

**Keywords:** Deuterium, Mitochondria, Rats, Tissues

## Abstract

**Background:**

This study aimed to evaluate the reaction of organism of laboratory animals on deuterium-depleted drinking diet. To assess the cell energy metabolism, the effect of a liquid medium with different deuterium contents on isolated liver mitochondria of random bred rats and Wistar rats was studied.

**Methods:**

This experimental study on the effect of deuterium-depleted drinking water (DDW) on 16-week-old male Wistar rats lasted for four weeks. Energy metabolism of mitochondria was examined through the production of hydrogen peroxide using an Amplex® Red Hydrogen Peroxide/Peroxidase Assay Kit.

**Results:**

Modification of isotope (deuterium-protium [D/H]) composition of rats’ blood and organ tissues with DDW (-705‰), introduced into rats’ diet within four weeks, led to the formation of isotope D/H gradient between blood plasma and organ tissues and affected isotope D/H exchange reactions on the adaptive processes. The isolated liver mitochondria from the random bred rats consumed DDW presented a maximum increase in H_2_O_2 _production during the incubation in DDW medium. This increased level of H_2_O_2_ production was higher in the isolated liver mitochondria of the rats consuming natural deuterium content drinking water (-24‰).

**Conclusion:**

The obtained results indicate the possibility of nutritional correction of isotope D/H metabolism in blood by means of products with modified isotope composition, as well as the prospects of using isotope exchange reactions in case of imbalance in function of the body's defense systems in different generations of animals.

## INTRODUCTION

Thanks to modern development of technologies, it has become possible to obtain water with specified ratio of light and heavy isotopes, in particular, ^1^H/^2^H and ^16^О/^18^O^[^^1^^]^. Under natural conditions, such pure water (^1^H_2_^16^O) cannot exist; therefore, to obtain it, either a delicate multi-stage purification of natural water is carried out, or it is synthesized from ^1^H and ^16^O^[^^1^^,^^2^^]^. The consumption of deuterium-depleted water (DDW) leads to a change in deuterium-protium (D/H) ratio in body tissues due to isotope exchange reactions, mainly activated by entropy changes observed in the living system^[^^3^^]^. 

The reactions of isotope (D/H) exchange between the liquid consumed and blood plasma occur quickly enough^[^^4^^]^. This matter would be the reason for carrying out studies on stable isotopes of biogenic elements in biological fluids and tissues. Some examples are the investigation of migration routes of wild animals^[^^5^^]^ or finding out where a person is living^[^^6^^]^. According to Chesson and co-authors^[^^7^^]^, δ^2^H and δ^18^O indices in local tap water are very clearly correlated with the concentration of such isotopes in the body of a person living in a given area. 

A number of studies have pointed out the diverse effects of isotope exchange reactions on the functional activity of biological systems, their native properties and structural organization^[^^8^^-^^10^^]^. However, most of the biological effects involving changes in isotope composition of biogenic elements in the body are still under investigation, and the primary concern is the effect of lower (compared to natural) concentrations of heavy non-radioactive isotopes on living systems. The latter is often associated with the traditional explanation of kinetic isotope effects, which is based on the idea that increase in their intensity is proportional to the concentration of heavy isotopes. In this case, however, isotope effects are associated with a deliberate decrease in the concentration of heavy non-radioactive isotopes in comparison to their natural concentration^[11]^. According to data found in literature, among works describing changes in the proportion of isotopes of biogenic elements, those devoted to the effect of various deuterium concentrations (δD) on the body are most frequently found, which can be explained by more pronounced isotope differences in nuclear masses of ^1^H and ^2^H in comparison to corresponding indices of atomic weight ratio of stable isotopes of oxygen, carbon and nitrogen. However, despite the increasing number of studies on the impact of low δD product on living systems, these studies mainly focus on alterations in isotope D/H ratio in blood plasma^[^^12^^-^^14^^]^. These investigations are not often adequate to comparatively study δD in body tissues and fluids during the active consumption of water containing modified isotope composition with reduced deuterium content^[^^15^^]^.

In general, the effect of DDW consumption on isotope composition of tissues and morpho-functional indices in multicellular organisms have not been studied extensively; nevertheless, this area is of particular interest since studying the morpho-functional status is one of the most informative ways of tracing individual development of an organism, as well as its health condition, and consumption of DDW affects the adaptive capacity of the organism in different periods of ontogenesis.

It is very important to make right choice of the purpose of research that would allow to estimate properly the diversity of the effect of isotope exchange reactions produced on biological systems, which seems to be essential in such scientific work. In scientific literature, one can find studies of various unicellular and multicellular organisms^[^^16^^-^^18^^]^ that are often presented in a discrete form, without taking into account the genetic heterogeneity of the studied subjects.

It is known that the general body responsiveness of outbred rats, for example, Wistar rats, is significantly lower due to low genetic heterogeneity than that of random bred animals of the same species^[^^19^^,^^20^^]^. In this regard, studies of the body’s adaptive capabilities under the influence of certain ambient factors (such as isotope D/H composition of the surrounding medium) are advisable to be conducted in both outbred and random bred rats. These investigations would make it possible not only to evaluate some specific reactions observed in the process of the isotope gradient change (in Wistar rats) but also to determine the entire range of adaptive responses (in random bred rats) which is important for the identification of accompanying morpho-functional effects associated with changes in the isotope D/H composition of the water consumed^[^^21^^,^^22^^]^.

The aim of the present study was to study the effect of isotope D/H exchange reactions on biochemical processes, cell energy metabolism, and the morpho-functional state of outbred and random bred rats in case of DDW consumption. 

## MATERIALS AND METHODS

DDW was produced on a plant designed in Kuban State University (Russia) by using electrolytic decomposition method^[2]^, with the initial concentration of deuterium in the water produced to amount of -705‰ (hereinafter indicates deuterium content), which was the lowest possibly attainable deuterium depletion degree for the plant in use. The water was further mineralized to use in animal experiments. Mineralization of the prepared water was performed by the addition of salts to obtain a physiologically high-grade mineral composition identical to that of water with deuterium content of -705 and -24‰. As a result, mineral content of produced DDW was 314–382 mg/l, which included 144–180 mg of hydrocarbons, less than 1 mg of sulfates, 60–76 mg of chlorides, 6 mg of calcium, 3 mg of magnesium, 50–58 mg of sodium, and 50–58 mg of potassium. 

The concentration of deuterium in the produced water was determined by means of a JEOL JNM-ECA 400MHz pulsed nuclear magnetic resonance spectrometer^[^^23^^]^. To determine the isotope D/H composition, liver samples (3.0 ± 0.1 mg each) were preliminarily freeze-dried using a lyophilizer ("LS-1000"; Prointech, Russian Federation)^[^^24^^]^. To determine the isotope D/H composition of the rats’ liver tissues and blood plasma, a DELTA^plus^ mass spectrometer equipped with a peripheral H/Device (Finnigan, Germany) was used for sample preparation of liquid for isotope analysis of hydrogen. 

 The biochemical studies were carried out by means of an biochemical autoanalyzer Chem Well 2900 T (USA), using Spinreact reagents (Spain). In the present study, the plasma levels of liver function biochemical markers, including bilirubin, aspartate amino-transferase (AST), and alanine aminotransferase (ALT) (De Ritis Ratio: AST/ALT 100) were measured.

The animals were managed, fed, attended, manipulated and removed from the experiment in accordance with the International Recommendations (the code of ethics). The use of animals in the experiments was approved by an ethical expertise (the local ethical committee of "The Gorbatov's All-Russian Meat Research Institute” FSBSI (No. 5 of 07.12.2016). The rats were kept in standard vivarium conditions: temperature 20 ± 3 °С, humidity 48 ± 2%, day/night lighting mode (from 6.00 a.m. to 6 p.m./from 6 p.m. to 6.00 a.m.), and free access to water and food. The rats were placed in plastic cages (TECNIPLAST type IV S), no more than four rats in each^[^^25^^]^. Throughout the experiment, the animals consumed standard complete feed. The safety of the experimental animals in both groups was 100%, and their physical activity was monitored during the entire experiment.

The experiment that was conducted to study the effect of DDW on 16-week-old male Wistar rats (obtained from the FSBIS SCBMT FMBA lasted for four weeks. During the experiment, two groups were defined:

(1) Group 1W, intact rats (control, n = 8) that were kept under standard conditions during the entire experiment and consumed drinking water with natural deuterium content (-24‰) *ad libitum*.

(2) Group 2W, rats (n = 16) that were kept under standard conditions throughout the experiment and consumed DDW (–705‰) *ad libitum*.

Another experiment which lasted for four weeks was simultaneously carried out to study the effect of DDW on 20-week-old random bred Albino male rats. During the experiment, group 1A (control group) was arranged similar to group 1W and group 2A (test group) similar to group 2W.

On 28^th ^day, animals were sacrificed in a euthanasia chamber (Vet Tech Solutions, UK) by means of carbon dioxide. Weight measurements were carried out regularly before each feeding step on the 1^st^, 7^th^, and 14^th^ days of the experiment; on 28^th^ day of the experiment, the rats were also weighed, followed by liver and blood sampling. Based on the obtained data, the integral index of chronic intoxication (IICI) was subsequently calculated according to the following formula; the results were presented in conventional units.

IICI = (m_liver_/m_animal_) 100

To assess the cell energy metabolism, the effect of a liquid medium with different deuterium contents on isolated liver mitochondria of random bred rats and Wistar rats was studied. One half of the samples in groups 1W, 2W, 1A, and 2A were obtained with double-distilled water (-24‰) as the solvent, and the other half with DDW (-705‰).

To isolate the mitochondria, all the reagents were prepared in µQ 18.2 MOhm/cm water (LaboStar TWF 7, Germany). All the mitochondrial isolation steps were carried out on ice using a previously described method^[^^26^^]^ with some modifications as below (steps 1–4).

Step 1. The freshly extracted liver (3.0 ± 0.2 g) was immediately immersed in a special isolation buffer (30 ml)-0.25 M sucrose solution (pH 7.4) containing 0.001 M EDTA (ethylenediaminetetraacetate). The liver sample was then washed three times with (each time 2.5 minutes) the same buffer.

Step 2. The washed liver sample was ground in a Petri dish on ice with a cooled ceramic knife, removing the connective tissues. The resulting (primary) homogenate was again rinsed three times with the isolation buffer (for 2.5 minutes), replacing the used solution with fresh buffer. Then the tissue was manually ground with a ceramic tool, not allowing the temperature to exceed 3 °C. For this purpose, 30 ml of isolation buffer was added to the homogenate once and then it was gently ground for 40 seconds. Another 30 ml of isolation buffer was added to the resulting (secondary) homogenate. The resulting homogeneous mixture was aliquated to 1.5-ml Eppendorf tubes.

Precipitation was carried out at 600 g for 10 minutes, with cooling to 1–2 °C in a high-speed laboratory cooled centrifuge RS-6MC (Kyrgyzstan). Due to these manipulations, undisturbed cells, large cell fragments and nuclear fraction were removed. The supernatant was carefully transferred into test tubes and kept on ice. The pellet and supernatant residues from the centrifuge tubes were re-suspended, then transferred to a single homogenization vessel and re-homogenized manually on ice for 20 seconds in 25 ml of isolation medium. After this homogenization step, the samples were repeatedly centrifuged. The resulting homogeneous mixture was poured into plastic centrifuge tubes; precipitation was carried out at 600 g for 10 minutes, with cooling to a temperature ranging from 1 to 2 °C. The resulting supernatants were combined in one container with supernatant kept on ice.

Step 3. The next step was the precipitation of the mitochondria. For this purpose, the combined supernatant was centrifuged at 14000 g for 10 minutes, and cooled to 1–2 °C in a centrifuge (Hermle Z 36 K, Germany). The supernatant was removed with a micropipette, and the resulting pellets were re-suspended and combined with 1 ml of isolation buffer. Then, 30 ml of the buffer was added in small portions with gentle shaking and centrifuged at 14000 g for 10 minutes, with cooling to a temperature ranging from 0 to 2 °C. The supernatant was removed with a micropipette, and the resulting pellets were re-suspended in 0.25 M sucrose solution (1 ml) lacking EDTA. Then 30 ml of this solution was added in small portions with gentle shaking and centrifuged at 14000 g for 10 minutes, and cooled to 1–2 °C.

Step 4. The supernatant was carefully removed, and 0.5 ml of 0.25 M sucrose solution was carefully layered onto the mitochondria precipitate. The upper loose precipitation layer (about 1 mm thick) was washed off by gentle shaking and removing by a micropipette. The procedures described in Step 4 were repeated two more times. The resulting solid pellet was then re-suspended in 0.5 ml of 0.25 M sucrose solution (without EDTA). The resulting suspension of isolated mitochondria was immediately used to perform Step 5.

Step 5. The concentration of mitochondria was determined from the protein content by means of the bichinol technique using a standard Pierce ™ BSA Protein Assay Kit (USA) in its microplate version. The plate reader, Thermo Scientific Multiskan FC (USA), was used for the measurement.

The mitochondrial suspensions were divided into two equal parts. One part was reacted with 50 µM succinate as a substrate for mitochondrial enzymes, and for the second part, no succinate was introduced, and all the redox processes occurred due to the pool of metabolites accumulated in the cell organelles.

The suspension of isolated mitochondria was incubated at 37 °C for 15 minutes in a phosphate buffer (pH 7.4) in the presence of 50 µM succinate (sodium salt) or without succinate.

According to the studied groups of rats, the following variants of isolated mitochondria were obtained: Mitochondria 1W and Mitochondria 1A, isolated liver mitochondria of rats consumed water with natural deuterium content, incubated in an aqueous medium with deuterium concentration equal to -24‰ (normal concentration); Mitochondria 1Wsuccinate and Mitochondria 1Asuccinate , isolated liver mitochondria of the rats consumed water with natural deuterium concentration, incubated in the presence of 50 μM succinate in an aqueous medium with deuterium concentration equal to -24‰ (normal concentration); Mitochondria 2WA and Mitochondria 2AA, isolated liver mitochondria of the pre-adapted rats (group 2), further (before measuring) incubated in an aqueous medium with δD = -705‰; Mitochondria 2WAsuccinate and Mitochondria 2AAsuccinate, isolated liver mitochondria of the pre-adapted rats (group 2), further (before measuring) incubated in the presence of 50 μM succinate in an aqueous medium with δD = -705‰; Mitochondria 2WB and Mitochondria 2AB , isolated liver mitochondria of the pre-adapted rats (group 2), further (before measuring) incubated in an aqueous medium with δD = -24‰; Mitochondria 2WBsuccinate and Mitochondria 2ABsu, isolated liver mitochondria of the pre-adapted rats (group 2), further (before measuring) incubated in the presence of 50 μM succinate in an aqueous medium with δD = -24‰; 

The concentration of hydrogen peroxide in the incubation medium was measured using an Amplex® Red Hydrogen Peroxide/Peroxidase Assay Kit (USA). The technique is based on the reaction of Amplex® Red reagent with hydrogen peroxide in the presence of horseradish peroxidase in phosphate buffer medium with dimethylsulfuroxide to form colored resofurin^[^^27^^]^. The production of hydrogen peroxide by mitochondrial suspensions was measured after preliminary 30-minute incubation of the sample with Amplex® Red Hydrogen Peroxide/Peroxidase Assay Kit in the dark at 25 °C. Absorption was measured using Thermo Scientific Multiskan FC with λ = 560 nm.

To investigate the effect of DDW on the perinatal development of rats a 40-week lasting experiment was set. In this experiment, four groups with eight rats in each were defined: two males and six females (one male and three females in one cage):

(1) Group A_0__W, intact Wistar rats (F0) that were kept in standard conditions throughout the experiment and consumed natural water (-24‰) *ad libitum*; the animals born from those in group A_0__W formed the first generation (F1) of rats (group A_1__W); (2) Group B_0__W, Wistar rats (F0) that consumed DDW (-705‰) from the age of four months, eight weeks before mating and then throughout the entire experiment; the animals born from those in group B_0__W formed the first generation (F1) of rats (group B_1__W); (3) Group C_0__A, intact random bred rats (F0) that were kept in standard conditions and consumed natural drinking water (-24‰) *ad libitum*; later on, the animals born from those in group C_0__A formed the first generation (F1) of rats (group C_1__A); (4) Group D_0__A, random bred rats (F0) that received DDW (-705‰) from the age of four months, eight weeks before mating and then throughout the experiment; later on, the animals born from those in group D_0__A formed the first generation (F1) of rats (group D_1__A).

The animals were kept in standard vivarium conditions: temperature 20 ± 3 °С, humidity 48 ± 2%, day/night illumination mode (from 6.00 a.m. to 6.00 p.m./from 6.00 p.m. to 6.00 a.m.), free access to water and food. The rats were placed in plastic cages (TECNIPLAST type IV S) with no more than four rats in each cage. Throughout the experiment, the animals consumed standard concentrated complete feed in accordance with GOST R 50258-92.

In groups A_1__W and C_1__A, infant rats were also given drinking water *ad libitum* with natural deuterium concentration (-24‰), and those in groups B_1__W and D_1__A consumed DDW *ad libitum* (-705‰) throughout the ontogenesis. The infant rats from all the four groups were weighed during three weeks after their birth (on the 1^st^, 7^th^, 14^th^, and 21^th^ days) in order to evaluate the effect of isotope exchange (D/H) reactions on the weight gain.

The reliability of the differences in the mean values ​​(M) found between the groups was statistically evaluated using a nonparametric U-test (Mann-Whitney), the difference was considered reliable for *p* < 0.05.

## RESULTS

Rats in groups 1W and 2W showed no statistically significant changes in body weight (from day 1 to day 28) and liver weight (Table 1); however, increase in appetite and a 5% increase in feed intake in group 2W were observed; physical activity, micturition, and defecation were within the physiological norm. Biochemical indices in blood plasma (ALT, bilirubin, and De Ritis ratio) did not statistically differ in groups 1W and 2W. AST activity index in the blood plasma of rats in group 2W was 9% lower than the same index in rats in group 1W.

The content of deuterium in the blood plasma of the rats in group 2W became 6.2 times lower than that of group 1W. The content of deuterium in the liver of 2W rats became 1.6 times lower than that of 1W rats. The change in body weight did not differ statistically in groups 1W and 2W throughout the experiment (weight 

gain equaled 13% in group 1W and 10% in group 2W).

The rats in groups 1A and 2A showed no statistically significant differences in body weight on day one, whereas on the 7^th^ day of the experiment, a 6% decrease in body weight was observed in rats of group 2A (Table 1) compared to the first day of the experiment. On the other hand, rats in group 1A showed 3% weight gain. The difference in the body weight of rats in groups 1A and 2A (8%) was still observed on the 14^th^ and 28^th^ days of the experiment. Throughout the experiment, the physiological indices of the rats in groups 1A and 2A were within the normal range. The liver weight of rats in group 2A on day 28 was 18% lower than that of rats in group 1A, and AST and De Ritis ratio in rats in group 2A were 15 and 43% higher, respectively. No statistically significant differences in ALT activity and bilirubin concentration were observed in groups 1A and 2A.

**Table 1:** Biochemical and morphological indices in adult male Wistar and random bred rats consumed DDW

**Parameter**	**Wistar rats**		**Random bred rats**	
	**Group 1W** **(152 ppm)**	**Group 2W** **(46 ppm)**	**Group** ** 1A** **(δ** **D** ** = –24‰)**	**Group** ** 2A** **(δ** **D** ** = –705‰)**
m (1), g	320.6 ± 17.3	324.7 ± 15.6	201.3 ± 5.7	198.5 ± 8.6
m (7), g	334.9 ± 13.6	326.1 ± 29.8	207.2 ± 8.1	187.1 ± 9.4***
m (14), g	355.4 ± 21.3	349.3 ± 20.4	226.9 ± 12.5	208.4 ± 16.7**
m (28), g	362.8 ± 28.6	358.6 ± 24.5	247.6 ± 11.4	228.7 ± 15.9**
Liver weight, grams (28)	10.02 ± 0.74	9.67 ± 1.17	7.08 ± 0.56	5.73 ± 0.92***
Integral Index of Chronic Intoxication (liver, 28)	2.77 ± 0.24	2.72 ± 0.35	2.87 ± 0.32	2.53 ± 0.51
Aspartate aminotransferase (AST), u/l (28)	132.57 ± 13.39	120.43 ± 42.56	146.72 ± 13.26	168.29 ± 15.78***
Alanine aminotransferase (ALT), u/l (28)	39.74 ± 2.95	37.23 ± 3.91	42.53 ± 3.62	35.81 ± 8.60
De Ritis Ratio (AST/ALT) in unit fractions (28)	3.36 ± 0.47	3.29 ± 1.28	3.46 ± 0.23	4.94 ± 1.19***
Bilirubin, micromole/l	2.84 ± 0.53	2.66 ± 0.71	6.27 ± 2.76	5.98 ± 1.37
Deuterium concentration in blood plasma, ‰ (28)	-59.3 ± 3.1	-367.2 ± 17.8*	-61.4 ± 3.0	-374.7 ± 25.1***
Deuterium concentration in liver, ‰ (28)	-128.7 ± 8.0	-210.1 ± 15.4*	-134.6 ± 12.4	-207.2 ± 19.3***

The parentheses indicate the day (from the beginning of the experiment) on which the index was measured.

* *p* < 0.01 compared with group 1W,

** *p* < 0.05 in comparison with group 1A,

*** *p* < 0.01 in comparison with group 1A.

During the pathoanatomical examination of 1W, 2W, 1A, and 2A rats, no inflammatory liver changes were detected. The liver was homogeneous dark-red and elastic with no signs of pathology. The IICI index did not differ statistically in groups 1W and 2W, as well as in groups 1A and 2A, indicating that there was no chronic intoxication in rats when DDW was consumed.

**Fig. 1:**
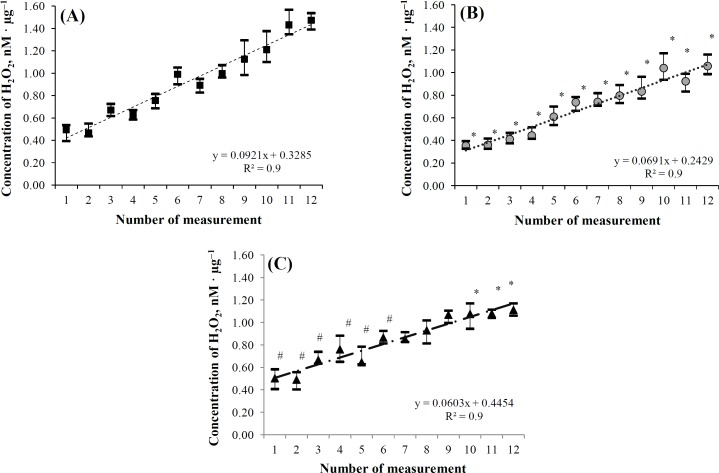
The dynamics of hydrogen peroxide specific production by isolated mitochondria of rat liver depending on the water consumption and the isotope composition of the water in the mitochondrial incubation medium. Concentration of H_2_O_2_ is  presented in nanomoles per microgram of mitochondrial protein (nM · µg^-1^). * *p* < 0.05 in comparison to mitochondria 2AA (1 is the number of measurement in 0 minutes from the beginning of the experiment, accordingly 2– in 10, 3– in 20, 4– in 30, 5– in 40, 6–in 50, 7– in 60, 8– in 70, 9– in 80, 10– in 90, 11– in 100, and 12– in 110 minutes); # *p* < 0.05 in comparison to mitochondria 1A

The content of deuterium in the blood plasma of rats in group 2A was 6.1 times lower than that of rats in group 1A. Deuterium content in the liver of 2A rats was 1.5 times lower than that of 1A rats, which indicates that the high ability of integral biological fluids to change their isotope composition depends on the content of deuterium in consumed water. The experiment also showed some differences in the dynamics of the specific production of hydrogen peroxide by the isolated liver mitochondria of random bred rats that had undergone preliminary adaptation. Their mitochondria were further incubated (before measurement) in aqueous media with δD = -705‰ (“mitochondria 2AA”) and δD = -24‰ (“mitochondria 2AB”), in order to study the stability of the preadaptation effect in the case of the changed isotope composition of the liquid medium (Fig. 1A and 1C). However, the isolated liver mitochondria of the rats consumed drinking water of natural deuterium content were incubated in an aqueous medium corresponding to a natural one (-24‰, "mitochondria 1A"), as shown in Figure 1B.

**Fig. 2:**
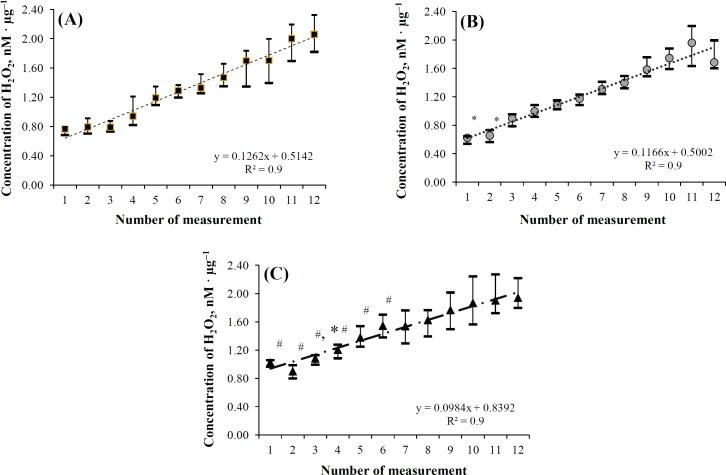
The dynamics of hydrogen peroxide specific production by isolated mitochondria of rat liver, depending on the water consumption, the isotope composition of the water in the mitochondrial incubation medium and the presence of succinate. The succinate concentration equaled 50 micromoles per liter. * *p* < 0.05 compared with mitochondria 2AAsu; # *p* < 0.05 compared with mitochondria 1Asu (1 is the number of measurement in 0 minutes from the beginning of the experiment, accordingly 2– in 10, 3– in 20, 4– in 30, 5– in 40, 6–in 50, 7– in 60, 8– in 70, 9– in 80, 10– in 90, 11– in 100, and 12– in 110 minutes). The concentration of H_2_O_2_ is presented in nanomoles per microgram of mitochondrial protein (nM · µg^-1^)

The isolated mitochondria of pre-adapted rats incubated in a medium with reduced deuterium content ("mitochondria 2AA") produced more hydrogen peroxide than isolated "mitochondria 1A" throughout the experiment (0 to 110^th^ minutes), with the maximum increase in the hydrogen peroxide produced by "mitochondria 2AA" by the 110^th^ minute being 35.3% (*p* = 0.0011) higher than that produced by the isolated "mitochondria 1A" (M ± σ = 1.068 ± 0.119 nM·µg^-1 ^of mitochondrial protein). Upon the addition of 50 μM succinate to the suspension of isolated mitochondria of groups 1A and 2A, the latter produced more hydrogen peroxide (Fig. 2). At the same time, the increase in the production of hydrogen peroxide by the 110^th^ minute in the "mitochondria 2AAsu" was 44.1% higher compared to "mitochondria 2AA" (M ± σ = 1.445 ± 0.129 nM·µg^-1 ^of mitochondrial protein). The increase in the production of hydrogen peroxide by the 110^th^ minute of the experiment in "mitochondria 1Asu" was 67.4% higher than that in "mitochondria 1A".

It was also notified that in the presence of 50 μM succinate in the suspension of "mitochondria 1Asuccinate" and "mitochondria 2AAsuccinate", differences in the production of hydrogen peroxide were observed from the beginning (0 min) till the 20^th^ minute of the experiment, reaching the maximum differences at the 20^th^ minute (M ± σ_«mitochobdria 1Asu»_ = 0.645 ± 0.1226 nM·µg^–^^1 ^of mitochondrial protein, M ± σ_«mitochondria 2AAsu»_ = 0.811 ± 0.149 nM·µg^–^^1 ^ of mitochondrial protein, *p* = 0.0357), whereas after that, no differences were observed up to the 110^th ^minute (*p* = 0.0742).

To check if the detected effect of *in vivo* pre-incubation in a medium with reduced deuterium concentration could be preserved, liver mitochondria of rats from group 2A were incubated in a medium with a natural deuterium concentration (-24‰). When the isolated liver mitochondria of pre-adapted rats were placed in the reaction system prepared on the basis of water with natural deuterium concentration (-24‰), an increased synthesis of hydrogen peroxide in the medium without succinate was observed during the first measurement: it was 36.8% (*p* = 0.0063) higher than that of "mitochondria 1A". Moreover, significant differences were observed till the 60^th^ minute of the experiment (Fig. 1B and 1C), then the differences were leveled and were not observed any more by the 110^th^ minute (*p* = 0.3446). Similar changes were observed when succinate was added with an ultimate concentration of 50 μM; the difference during the first measurement was 69.2% (*p* = 0.0008) and preserved till the 50^th^ minute of the experiment (Fig. 2B and 2C). From the 60^th^ minute, the differences elevated and were no longer observed after the 110^th^ minute of the experiment (*p* = 0.0929).

**Table 2:** The dynamics of body weight of the 1st generation (F1) random bred rats during the first three weeks of postnatal development

**Parameter**	**Group** **C**_1_**_А (–24‰)**	**Group** **D**_1_**_А (–705‰)**
m (1), g; group C_1__А (n=54) group D_1__А (n=37)	5.7 ± 0.8	5.5 ± 0.3
m (7), g; group C_1__А (n=47) group D_1__А (n=34)	10.1 ± 0.9	8.4 ± 1.0 *
m (14), g; group C_1__А (n=46) group D_1__А (n=34)	13.6 ± 1.7	13.5 ± 1.2
m (21), g; group C_1__А (n=46) group D_1__А (n=34)	17.3 ± 1.6	19.2 ± 1.5 *

* *p* < 0.05 compared to group C_1__A. The parentheses indicate the day of the experiment, on which body weight was measured.

The mathematical model, most closely described the results of hydrogen peroxide production under the given measurement conditions in isolated mitochondria of rat liver, is a linear model with the following type of equation: Y = kX + b. The coefficient of approximation reliability (R^2^) for the presented curves exceeded 0.93 (Figs. 1 and 2), which confirms the correspondence of the obtained linear model to the initial experimental data. Higher energy metabolism in mitochondria could be manifested by a higher body weight gain in random bred infant rats in F1, which was established during the study of their body weight dynamics in postnatal period (Table 2).

During the first days after birth, a less pronounced body weight gain in the first-generation infant rats in group D_1__A was observed with a 17% increase, reaching the greatest difference compared to C_1__A group by the end of the first week. On the first day, the number of newborn rats in C_1__A group was 46% more than in the group of rats that were given DDW. However, the dispersion index (root mean square deviation) of body weight on day one in group D_1__A was 63% lower than in group C_1__A, which indicates reduction in the difference of body weight during a decrease in the number of rats in the litter under the conditions of DDW consumption. Later, at the end of the 2^nd^ week, the dynamics of body weight gain accelerated in the rats of D_1__A group, while the absolute values ​​of the morphological parameters reached the control group C_1__A on the 14^th^ day (*p* > 0.05) and significantly exceeded the rat body weight of this group by 11% on the 21^st^ day after birth.

Apparently, the change in the isotopic D/H gradient indirectly affected pregnant females, causing the birth of a larger percentage of viable rats with less body mass dispersion, due to more development of a smaller number of embryos in the uterus of random bred rats. Thus, on the 7^th^ day, the percentage of dead F1 rats in group D_1__А was 8%, 1.6 times less than the number of dead rats in group C_1__А that had consumed drinking water of natural deuterium content, although the absolute number of surviving animals in group C_1__А on the 7^th^ day was 38% higher than that in group D_1__A. On 14^th^ day, the number of surviving rats in group D_1__A did not change in comparison to that of the 7^th^ day, whereas in group C_1__А, the number of surviving rats was 98% on the 7^th^ day.

In the case of Wistar rats, no significant differences was observed in the production of hydrogen peroxide between the groups "mitochondria 1W", "mitochondria 2WA", and "mitochondria 2WB". There were also no statistically significant differences between the indices of hydrogen peroxide production in groups “1Wsuccinate mitochondria”, “2WAsuccinate mitochondria”, and “2WBsuccinate mitochondria” (*p* ˃ 0.05). No statistically significant difference was observed in the body weight of the Wistar rats of F1 generation (Table 3).

During the first seven days, a greater percentage (79%) of deaths was recorded among infant rats in the F1 litter of the Wistar rats had been consuming DDW compared to group А_1__W; the percentage of dead rats was 10%. This observation may indicate a stressful effect produced by the changing isotopic D/H gradient on pregnant females, causing the birth of a significant number of nonviable rats with no differences in morphometric characteristics observed between these rats and the intact ones. Moreover, on the 14^th^ day, the number of surviving infant rats in F1 was 77% in group В_1__W, whereas the number of surviving infant rats in group А_1__W was 14% higher, which indicates a less stressful effect produced on the young rats in the case of consumption of water with natural deuterium content by the pregnant females. In this case, the absolute number of surviving rats (53 and 55) on day 14 in groups А_1__W and В_1__W was almost equal.

**Table 3:** The dynamics of body weight gain of the 1st generation (F1) Wistar rats during the first three weeks of postnatal development

**Parameter**	**Group** ** A**_1_ **_W** ** (** **–24‰** **)**	**Group** ** В**_1_ **_W** ** (** **–705‰** **)**
m (1), g; group A_1__W (n=59) group В_1__W (n=71)	7.1 ± 0.8	6.9 ± 1.0
m (7), g; group A_1__W (n=53) group В_1__W (n=58)	14.4 ± 1.3	14.7 ± 1.8
m (14), g; group A_1__W (n=53) group В_1__W (n=55)	23.8 ± 2.4	26.3 ± 3.1
m (21), g; group A_1__W (n=53) group В_1__W (n=55)	46.5 ± 2.6	47.1 ± 4.3

The parentheses indicate the day of the experiment on which body weight was measured.

## DISCUSSION

The analysis of fluctuations of the isotope D/H composition of lyophilized liver tissues showed that small changes in deuterium concentration in the liver may exhibit the presence of sufficiently selective histohematological barriers, restricting to some extents to the amount of heavy isotopes entering hepatocytes and also probably to the formation of intracellular water from alternative substrates with more stable isotope composition, for example, in tricarboxylic acid cycle^[^^28^^]^*.*

More rapid changes in deuterium concentration in blood plasma compared to fluctuations in its concentration in liver tissues cause a change in the direction and magnitude of isotope D/H gradient, which under physiological conditions is characterized by the following relation: (δD_plasma_/δD_tissue_) ˃ 1. However, in group 2A rats, the physiological direction of the gradient changed in opposite direction ([δD_plasma_/δD_tissue_] ˂ 1).

Such uneven fluctuations in the isotope D/H composition of tissues and biological fluids can be characterized by changes in the functional status of the organism due to the development of general nonspecific adaptive reactions that develop in response to the action of any endogenous or exogenous factor*.*

The uneven decrease in deuterium concentration in the rats’ liver and blood plasma can indicate the formation of isotope gradient due to faster reduction of deuterium content in the blood, and the change in the rats’ body weight can characterize the process of adaptation of the organism to changing living conditions. The direct influence of DDW on morphofunctional indices and biochemical processes of the organism, in addition to the phenomenon of preliminary adaptation, can also be associated with the enhancement of isotope exchange (D/H) in active and allosteric sites of enzymes. It is known that D/H isotope exchange reactions are mostly active in compounds consisting of atoms with an unshared electron pair and capable of forming intermediate metabolic complexes with the participation of hydrogen bonds, in which protons (H^+^) and deuterons (D^+^) simultaneously move from one molecule to another. The relatively selective substitution of deuterium for protium in active sites of enzymes is explained by the fact that the hydrogen exchange between water and various hydrogen-containing entities under physiological conditions occurs quite easily only in those A–H bonds, where the atom (A = O, S, N) contains a free electron pair, or between compounds in a response complex formed via a hydrogen bond. Under natural conditions, there is no exchange in the hydrocarbon linkage R_3_С–Н(D) because the carbon atom does not have a free electron pair. Such selectivity of isotope exchange shows that even an insignificant isotope gradient can produce a selective effect on metabolically active compounds containing the largest number of active atoms having an unshared electron pair and capable of forming hydrogen bonds. On the other hand, a decrease in deuterium content in the hydration shell is also accompanied by changes in the biological activity of macromolecules, which is due to higher frequency and amplitude of vibrations of atomic groupings consisting only of light isotopes. Such rearrangements of biochemical processes in tissues with high metabolic activity (such as in the liver) can affect the morphofunctional indices of the whole organism, for instance, in rats in group 2A. At the same time, no similar changes in morphological parameters were observed in group 2W during the experiment, which may indicate a narrower range of adaptive reactions when the isotope gradient is changed in Wistar rats (group 2W), unlike random bred rats (group 2A) apparently having a wider range of adaptive reactions.

Fluctuations in the isotope composition of tissues can lead to an increase in the activity of nonspecific defense systems, which is explained by the phenomenon of preliminary adaptation potentiating the defense mechanisms in cells under stressful conditions caused by various factors (for example, temperature, hypoxia, etc.). A non-specific defense system can react according to a similar mechanism under the conditions of created D/H gradient, which may be due to the response of cellular regulatory systems, taking into consideration that a living matter gravitates toward the constancy of the isotope composition corresponding to that of the natural habitat of a biological object. Adaptive responses can also be realized due to a more active deuterium exchange in transcription factors, as well as in the hydration shell of proteins and nucleic acids, which can change their thermodynamic and consequently, thermokinetic characteristics, thus increasing the adaptive capacity of the organism even in the case of relatively insignificant (from 10 to 30%) exchange of heavy isotopes in tissues. A slightly expressed and slower D/H exchange in organs is possible due to the additional amount of deuterium consumed with nutrients.

In order to study the mechanisms of the influence of D/H gradient on energy metabolism in the liver, the dynamics of hydrogen peroxide production by isolated mitochondria of rat liver were studied, depending on whether the rats underwent preadaptation to reduced concentration of deuterium in drinking water (δD = -705‰) *in vivo* during four weeks and incubation *in vitro* in a medium with reduced deuterium content (δD = -705‰). The mechanism of the biological effect of the changed ratio of stable isotopes of hydrogen (D/H) is connected with the fact that a liquid medium with reduced deuterium content is directly interrelated with the tricarboxylic acid cycle. This is due to the fact that the enzymes of the tricarboxylic acid cycle localized in the inner mitochondrial volume provide redistribution of deuterium between cytoplasmic and mitochondrial water pools^[^^29^^]^. In this case, decrease of δD in cytoplasmic water contributes to the normalization of the phenotype of cells with unbalanced regulation of metabolism. This scenario leads, for example, to a decrease in cancer cells division rate^[^^30^^]^.

The preliminary adaptation of rats to a medium with low deuterium content was a part of the present experiment, since it is known that some proton-active enzymes (such as ATPase-dependent proton (^1^H^+^) pump) show selectivity for stable hydrogen isotopes. In particular, they are inhibited to different extents by deuterium^[^^31^^]^.

The production of hydrogen peroxide is associated with oxidation-reduction reactions involving energy exchange in the mitochondria. Therefore, the increased production of hydrogen peroxide by mitochondria adapted to a medium with reduced deuterium content may indicate the ability of such mitochondria to synthesize more ATP or other macroergs, which indicates the presence of conditions for the activation of anabolic processes in the body. It is known that the production of hydrogen peroxide by the mitochondria is associated with the formation of reactive oxygen intermediates formed as a result of electron leakage from some components of the respiratory chain^[^^16^^]^, and other processes, including the active cycle of tricarboxylic acids. Therefore, since there is no separation of oxidative phosphorylation, the increased production of hydrogen peroxide can be considered as a positive indicator of the effective work of the mitochondrial energy system.

It is known that the generation of hydrogen peroxide by mitochondria in the presence of succinate is a process that is extremely sensitive to the effectiveness of transmembrane proton transfer agents^[^^32^^]^. This study showed that in the presence of succinate at 50 μM concentration, the differences observed in the medium with low deuterium content gradually elevated. This reduction in the medium may be related to the significantly greater influence of succinate (as the reaction substrate) on the rate of redox reactions of the Krebs cycle compared to the effects caused by the formation of deuterium transmembrane gradient in the mitochondria. Thus, it has been shown that the character of the dependence of hydrogen peroxide production on the initial D/H ratio within mitochondria is determined by the presence of succinate– one of the intermediates in the tricarboxylic acid cycle– and other metabolic processes in the mitochondria. Similar effects, for instance, those of water with reduced δD, may be ascribed to the fact that its consumption increases the production of macroergic compounds in cells (including adenosine triphosphate) and the energy resources of the whole organism during metabolic adaptation at the cellular level^[^^33^^]^, which can have a significant effect on the body due to a decrease in intracellular δD^[^^33^^,^^34^^]^. This matter provides further increase in the rate of reducing reactions involving the deuterium-free form of the reduced nicotinamide adenine dinucleotide phosphate (NADPH) that is necessary for the synthesis of various signal molecules ^[35]^. At the same time, the reduction of deuterium content in a DNA molecule, according to some authors, contributes to the stabilization of its native structure and reduces the risk of replication and transcription failure due to more functional (energetically favorable) protium hydrogen bonds^[^^36^^]^. Therefore, a disturbance in mitochondria functioning due to the effect produced by hypoxia, acidosis, or other pathogenic factors on the cell can reduce the content of the deuterium-free form of the reduced NADPH, hence reducing the transfer of ^1^H (H-transfer occurring in the transhydrogenase reaction) to NADPH. It is possible that these phenomena may be caused by the changes in the transfer of ^1^H atoms formed in the mitochondria via beta oxidation of fatty acids characterized by a lower deuterium content^[^^37^^-^^39^^]^. In addition, ^2^H atoms, the concentration of which is higher in the water and glucose coming to the cytosol from the extracellular medium^[^^40^^,^^41^^]^, leads in general to an increase in the intracellular D/H ratio. Higher values ​​of this ratio can result in a change in the rate, and more rarely, to a change in the direction of biochemical reactions in the body, creating metabolic prerequisites for reducing adaptation and body weight. Thus, in cells with mitochondria with disrupted transfer of ^1^H atoms, it is possible to regulate the production of macroergic compounds by decreasing the concentration of deuterium in the water consumed, which can correct the growth of cells, their division, and functional disorders^[^^42^^]^.

Based on the obtained data, the following conclusions were drawn: in all mitochondrial samples, linear accumulation of hydrogen peroxide was observed throughout the entire duration of the experiment, i.e. for 110 minutes (with 10-minute periodicity of measurements). Apparently, the reactions of isotope D/H exchange in the rats of F1 generation lead to a less pronounced increase in body weight during the first week after their birth. However, later during the third week, the dynamics of growth in the rats of F1 generation exceeded that of the control group, which reflects a faster increase in the long-term adaptive capacity of the organism in the case of reduced deuterium content throughout the ontogenesis, including the antenatal period. The obtained results allow us to speak about the ability of water with reduced deuterium content to increase the potential of the organism defense system during preparing it for subsequent stressful effects or in case of potential development of alternative pathological processes. In general, the obtained results indicate the possibility of nutritional correction of isotope D/H exchange in blood by means of products with a modified isotope composition, as well as the prospects of using isotope D/H exchange reactions in case of imbalanced function of the body's defense systems^[^^43^^]^. All these results can be related to a significantly lower limit of adaptive reactions realization, compared to random bred rats with greater genetic heterogeneity, which allows the latter to implement their individual adaptive capabilities more effectively when facing adverse environmental triggers. However, similar mechanisms of adaptive reactions under stress conditions are accompanied by a decrease in animals’ breeding performance, with the formation of a higher adaptive potential in surviving offspring in the postnatal period of its development, which is characterized by greater individual resistance of these rats to environmental effects. Meanwhile, Wistar rats in group В_1__W, due to a lower degree of implementation of adaptive reactions to the changing isotopic D/H gradient in the females during the pregnancy period, gave birth to a greater number of rats (92% more compared to group D_1__А), which demonstrated less ability to adapt to environmental effects.

In general, the obtained results indicate the possibility of nutritional correction of isotope D/H metabolism in the blood by means of products with modified isotope composition, as well as the prospects of using isotope exchange reactions in case of imbalance in the work of the body's defense systems in various generations of animals.
